# A New Two-Stage Algorithm for Solving Optimization Problems

**DOI:** 10.3390/e23040491

**Published:** 2021-04-20

**Authors:** Sajjad Amiri Doumari, Hadi Givi, Mohammad Dehghani, Zeinab Montazeri, Victor Leiva, Josep M. Guerrero

**Affiliations:** 1 Department of Mathematics and Computer Science, Sirjan University of Technology, Sirjan, Iran; amiri@sirjantech.ac.ir; 2 Department of Electrical Engineering, Shahreza Campus, University of Isfahan, Iran; h.givi@shr.ui.ac.ir; 3 Department of Electrical and Electronics Engineering, Shiraz University of Technology, Shiraz, Iran; adanbax@gmail.com (M.D.); z.montazeri@sutech.ac.ir (Z.M.); 4 School of Industrial Engineering, Pontificia Universidad Católica de Valparaíso, Valparaíso 2362807, Chile; 5 CROM Center for Research on Microgrids, Department of Energy Technology, Aalborg University, 9220 Aalborg, Denmark; joz@et.aau.dk

**Keywords:** Friedman test, machine learning, population-based optimization, swarm intelligence

## Abstract

Optimization seeks to find inputs for an objective function that result in a maximum or minimum. Optimization methods are divided into exact and approximate (algorithms). Several optimization algorithms imitate natural phenomena, laws of physics, and behavior of living organisms. Optimization based on algorithms is the challenge that underlies machine learning, from logistic regression to training neural networks for artificial intelligence. In this paper, a new algorithm called two-stage optimization (TSO) is proposed. The TSO algorithm updates population members in two steps at each iteration. For this purpose, a group of good population members is selected and then two members of this group are randomly used to update the position of each of them. This update is based on the first selected good member at the first stage, and on the second selected good member at the second stage. We describe the stages of the TSO algorithm and model them mathematically. Performance of the TSO algorithm is evaluated for twenty-three standard objective functions. In order to compare the optimization results of the TSO algorithm, eight other competing algorithms are considered, including genetic, gravitational search, grey wolf, marine predators, particle swarm, teaching-learning-based, tunicate swarm, and whale approaches. The numerical results show that the new algorithm is superior and more competitive in solving optimization problems when compared with other algorithms.

## 1. Introduction

Optimization is the science of finding the best solution available for a problem, maximizing or minimizing the corresponding objective function. Each optimization problem has essentially three elements: (i) decision variables; (ii) objective function; and (iii) constraints. An optimization problem can have more than a solution, reason why its global optimum is called the main solution [1].

Methods to solve optimization problems may be divided into two categories: (i) exact and (ii) approximate [2]. Exact methods are able to find the optimum accurately, but they are not efficient enough in complex problems, with their execution times being increasing exponentially according to the problem dimension. The approximate methods (or algorithms) are able to find good (near-optimal) solutions in a short time for complex problems.

There are numerous optimization problems in engineering and sciences that can be solved with different algorithms, where the population-based approaches are often considered as one of the most effective methods in solving such problems [3]. Note that optimization is the challenging problem that underlies many machine and statistical learning algorithms, from the logistic regression model to training artificial neural networks, tools which are fundamental for the development of artificial intelligence [4].

In order to optimize the objective function, population-based algorithms are able to find appropriate values for the decision variables, based on the constraints to which this function is subject to, through random scanning of the problem search space [5].

Although optimization algorithms provide good solutions, they do not necessarily attain the global optimum. However, often these solutions are close to this optimum and then accepted as a quasi-optimal solution. In order to evaluate the performance of the approximate methods in solving optimization problems, an algorithm is superior to another if the former one provides a better quasi-optimal solution than the last one. 

Some researchers have focused on designing algorithms to provide quasi-optimal solutions closer to the global optimum. In this regard, diverse algorithms have been applied by engineers and scientists in various fields such as engineering [6] and energy [7] to achieve quasi-optimal solutions.

Therefore, mainly in computationally highly complex and challenging optimization problems, different practitioners are interested on improving the computational efficiency of the algorithm used to solve such problems. Consequently, population-based algorithms can be useful to deal with this improvement considering two stages of updating of population members. To the best of our knowledge, this two-stage approach has not been until now considered to improve population-based algorithms.

The main objective of this paper is to propose a new algorithm called two-stage optimization (TSO). The TSO algorithm updates each population member in two stages based on a selected group of the good members. Accordingly, the position of a member of the population is updated using two randomly selected members of the good group.

The rest of the article is organized as follows. Section 2 provides an overview of optimization algorithms published in the literature, mentioning several related works. Then, in Section 3, the proposed TSO algorithm is introduced. The performance of the new algorithm in solving optimization problems is evaluated in Section 4. We present further analysis of the results and discussion on the performance of the TSO algorithm in Section 5. Finally, conclusions and suggestions for future works are given in Section 6.

## 2. Literature Review

In this section, we provide an overview of optimization algorithms published in the literature.

The main purpose of the algorithms is to search effectively and efficiently for the solution space of the optimization problem, as well as to apply rules and strategies to guide the search process. In population-based optimization algorithms [3], a population of random solutions is created first [5]. Then, in an iterative process, this population is improved using rules of the algorithm. The principal idea of the population-based algorithms is to update the population in successive iterations, providing better quasi-optimal solutions. An optimization algorithm may provide a reasonable solution to some problems but inadequate to others. Therefore, the main indicator to compare the performance of optimization algorithms is the value of the objective function.

Optimization algorithms have been inspired by various natural phenomena, behavior of living organisms, plant growth, physical laws, and rules of the games, among others. In general, this type of algorithms can be classified into four groups including: (i) evolutionary-based, (ii) game-based, (iii) physics-based, and (iv) swarm-based approaches, which are detailed below.

Evolutionary optimization algorithms [8] were derived by taking into account genetic processes, especially reproduction. Genetic algorithms [9] are the most famous and widely used of this group, which are based on simulating the birth process and Darwin theory of evolution. In these algorithms, population members are updated based on: (i) selection, (ii) crossover, and (iii) mutation. The differential evolution [8] is proposed to overcome the drawback of the genetic algorithm [9], namely its lack of local search. The main difference between the genetic algorithm and differential evolution is in the selection operators. For these operators of the genetic algorithm, the chance of selecting an answer as one of the parents depends on the value of its objective function, but in the differential evolution all answers have an equal chance of being selected. Therefore, this chance does not depend on the value of its objective function. The artificial immune system evolutionary algorithm is inspired by the mechanisms of the human body and designed by simulating the defense mechanism against disease, microbes, and viruses [10].

Game-based algorithms [11] are developed by simulating the rules of various individual and group games with the aim of solving optimization problems. The orientation search is one of the algorithms in this group, which has been designed by considering the orientation game rule. With this rule, the players move on the playground (that is the same as search space) according to the direction indicated by the referee. Football game-based optimization is another of these algorithms which is formulated by simulating the behaviors and policies of clubs in the football league. In this algorithm, the population is updated in four phases: (i) holding the league, (ii) training the clubs, (iii) transferring the players, and (iv) relegation and promotion of the clubs [12].

Swarm-based optimization algorithms [13] are widely considered and designed mimicking the behaviors of animals, plants, and living organisms, as well as other population-based phenomena [14]. One of the most famous algorithms is the particle swarm optimization (PSO), which imitates the birds’ movement. The process of population updating in the PSO algorithm [15] is based on individual knowledge (local best) and the knowledge of the whole population (global best). Teaching-learning-based optimization (TLBO) is another algorithm in this swarm-based group that was introduced following the teaching-learning process between students and teacher [16]. Grey wolf optimization is also in the group of swarm intelligence algorithms and is inspired by nature. This algorithm simulates the hierarchical structure of social behavior of gray wolfs during hunting [17]. When implementing the algorithm, four types of gray wolf (alpha, beta, delta, and omega) are used to model their hierarchical leadership, with three hunting steps being executed: (i) search for prey, (ii) siege of prey, and (iii) attack on prey. The marine predators (MP) algorithm is inspired by the movement strategies that marine predators use when trapping their prey [18]. In the first phase, MP generates a random population of predators in the search space. Then, given that stronger hunters get more chances and share of food, the best solution is applied. Tunicate swarm (TS) is an optimization algorithm that imitates the jet propulsion and swarm behaviors of tunicates during the navigation and foraging process [19]. Whale optimization (WO) is an algorithm inspired by the bubble net hunting method of whales [20]. The WO is performed into three phases: (i) encircling prey, (ii) bubble-net attack, and (iii) searching for prey.

Physics-based algorithms are designed using physical laws to achieve quasi-optimal solutions [21]. One of these optimizers is the gravitational search (GS), which was formulated by simulating the law of gravitational force between objects [22]. Simulation of the Hooke and spring displacement laws were applied to designing the spring search algorithm [23]. In this algorithm, population members correspond to weights connected to each other by different springs. These members are updated moving in the search space using the forces exerted on the weights by the springs. The Henry gas solubility algorithm tries to imitate the behavior governed by the Henry law to solve challenging optimization problems. This is an essential law that states that the amount of gas dissolved in a liquid is proportional to its partial pressure on the liquid at a fixed temperature. The Henry algorithm imitates the huddling behavior of gas to balance exploration and exploitation in the search space and avoid local optima [24].

## 3. The New Two-Stage Optimization Algorithm

In this section, the stages of the proposed TSO algorithm are described and then mathematically modeled to be implemented on various optimization problems.

### 3.1. Theory of the TSO Algorithm

In most population-based optimization algorithms, the member that provides the best value of the objective function (the best member) has an impressive impact on population update and algorithm progress. However, the position of the best member in the problem search space may not be appropriate in all axes (decision variables). This concept means that the best member might not be suitable for leading the population in some axes.

The main idea of the TSO algorithm for solving such an issue is to employ a selected group of good members of the population called the good group. The use of this group in population updating utilizes more information in population development to achieve a quasi-optimal solution. Each member in the TSO algorithm is updated in two stages. At each stage of this algorithm, a member of the good group is randomly selected to update the position of each population member on each axis of the search space. This population update continues iterating until the algorithm stops. Then, when the algorithm reaches the stopping condition, the best quasi-optimal solution for the problem is reported. In the next subsection, mathematical modeling of the TSO algorithm is presented.

### 3.2. Mathematical Modeling of the TSO Algorithm

As mentioned, the TSO algorithm is a population-based optimization technique. Each row of the population matrix belongs to a population member, which proposes values for the decision variables. Each column of this matrix also specifies values of a variable proposed by different members. Therefore, for the population matrix, the number of rows is equal to the number of members, whereas the number of columns is equal to the number of decision variables. The population matrix (X) of the TSO algorithm is defined as

$$X=\left[\begin{array}{c} \vec{X_{1}}\\ \vdots \\ \vec{X_{i}}\\ \vdots \\ \vec{X_{N}} \end{array}\right]={\left[\;\begin{array}{ccccc} x_{1}^{1} & \cdots & x_{1}^{d} & \cdots & x_{1}^{m}\\ \vdots & \ddots & \vdots & ⋰ & \vdots \\ x_{i}^{1} & \cdots & x_{i}^{d} & \cdots & x_{i}^{m}\\ \vdots & ⋰ & \vdots & \ddots & \vdots \\ x_{N}^{1} & \cdots & x_{N}^{d} & \cdots & x_{N}^{m} \end{array}\;\right]}_{N\times m},$$

where $\vec{X_{i}}$ is the *i*’-th population member, $x_{i}^{d}$ is the suggested value for the *d*’-th variable by the *i*’-th population member, m is the number of variables, and N is the number of members. After defining the mentioned matrix, the objective function is evaluated based on the corresponding members according to the values proposed for the variables. By comparing the obtained values, a certain number of population members (for example, a ten percent), for which quasi-optimal values have been achieved in the objective function, are selected as members of the good group. This group is described using the matrix representation stated as

$$G=\left[\begin{array}{c} \vec{G_{1}}\\ \vdots \\ \vec{G_{j}}\\ \vdots \\ \vec{G_{N_{G}}} \end{array}\right]={\left[\;\begin{array}{ccccc} g_{1}^{1} & \cdots & g_{1}^{d} & \cdots & g_{1}^{m}\\ \vdots & \ddots & \vdots & ⋰ & \vdots \\ g_{j}^{1} & \cdots & g_{j}^{d} & \cdots & g_{j}^{m}\\ \vdots & ⋰ & \vdots & \ddots & \vdots \\ g_{N_{G}}^{1} & \cdots & g_{N_{G}}^{d} & \cdots & g_{N_{G}}^{m} \end{array}\;\right]}_{N_{G}\times m},$$

where $\vec{G_{j}}$ is the *j*’-th good member, $g_{j}^{d}$ is the *d*’-th dimension of the *j*’-th good member, and $N_{G}$ is the number of selected good members. The main idea in the TSO algorithm is to update the value of each variable (proposed by each member of the population) using two different members of the good group.

In the first stage, the position of each population member on each axis of the search space is updated with a selected good member. Thus, a good member may be selected to lead a population member on one or more axes. In addition, a good member may not be selected to lead other members on any of the axes. The first stage of the TSO algorithm for updating population members is expressed as

(1) $${x^{\prime }}_{i}^{d}=\{\begin{array}{l} x_{i}^{d}+\mathrm{rand}\times \left(g_{j}^{d}-x_{i}^{d}\right),\;F_{j}<F_{i},\\ x_{i}^{d}+\mathrm{rand}\times \left(x_{i}^{d}-g_{j}^{d}\right),\;\mathrm{else}; \end{array}\;j\in 1:N_{G},$$

(2) $$\vec{X_{i}}=\{\begin{array}{c} \vec{{X^{\prime }}_{i}},\;{F^{\prime }}_{i}<F_{i,}\\ \vec{X_{i}},\;\mathrm{else}, \end{array}$$

where ${x^{\prime }}_{i}^{d}$ is the new position of the *i*’-th member in the *d*’th dimension, rand is a random number in the interval [0, 1], $F_{i}$ is the value of the objective function for the *i*’-th population member, $\vec{{X^{\prime }}_{i}}$ is the new position of the *i*’-th member, and ${F^{\prime }}_{i}$ is its corresponding objective function value. Equation (1) indicates that a member is updated if the value of the objective function is improved in the new position.

In the second stage, the position of each member, on each axis of the search space, is updated again based on a non-repetitive good member. This means that the position of each member, on each axis, is affected by two different members of the good group. This stage of the TSO algorithm in updating population members is defined as

(3) $${x^{\prime }}_{i}^{d}=\{\begin{array}{l} x_{i}^{d}+\mathrm{rand}\times \left(g_{k}^{d}-x_{i}^{d}\right),\;F_{k}<F_{i},\\ x_{i}^{d}+\mathrm{rand}\times \left(x_{i}^{d}-g_{k}^{d}\right),\;\mathrm{else}; \end{array}\;k\in 1:N_{G},\;k\neq j,$$

(4) $$\vec{X_{i}}=\{\begin{array}{c} \vec{{X^{\prime }}_{i}},\;{F^{\prime }}_{i}<F_{i,}\\ \vec{X_{i}},\;\mathrm{else}. \end{array}$$

**Figure 1:**
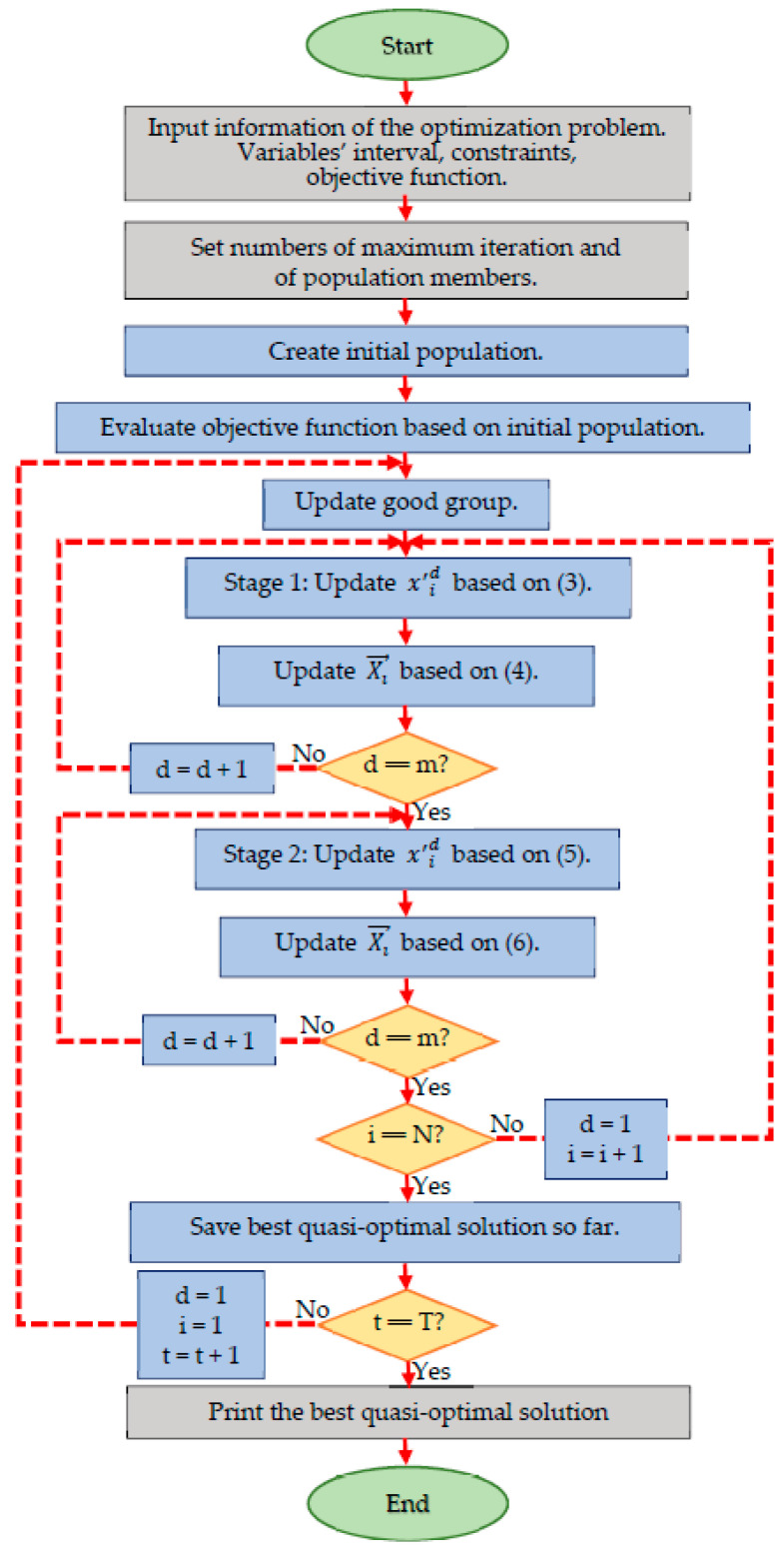
Flowchart of the TSO algorithm.

After updating the population based on the mentioned two stages, new members of the good group are selected. This process is repeated until the algorithm reaches the condition of stopping. The implementation process of the TSO algorithm is presented as a pseudo-code in Algorithm 1. Furthermore, the steps of the TSO algorithm are shown as a flowchart in Figure 1.
**Algorithm 1** Pseudo-code of the TSO approach.Start the TSO algorithm.1.Determine the range of decision variables, constraints and objective function of the problem.2.Create the initial population at random.3.Evaluate the objective function based on the initial population.4.  For *t* = 1:*T*, with *t* being iteration number and *T* the maximum iteration:5.  Update the good group.
6.      For *i* = 1:*N*, with *N* being the number of population members;7.          For *d* = 1:*m*, with *d* being the contour and *m* the number of variables:8.              Select the *j’-*th good member.9.              Stage 1: Update ${x^{\prime }}_{i}^{d}$ based on (1).10.              End for *d* = 1:*m*.11.          Update $\vec{X_{i}}$ based on (2).12.          For *d* = 1:*m*:13.              Select the *k’-*th good member, with *k* ≠ *j*.14.              Stage 2: Update ${x^{\prime }}_{i}^{d}$ based on (3).15.          End for *d* = 1:*m*.16.          Update $\vec{X_{i}}$ based on (4).17.      End for *i* = 1:*N*.18.  Save the best quasi-optimal solution.
19.  End for *t* = 1:*T*.20.  Print the best quasi-optimal solution obtained by the TSO algorithm.End the TSO algorithm.

## 4. Simulation Study and Results

In this section, the performance of the TSO algorithm for solving optimization problems is evaluated. For this purpose, the algorithm has been implemented on twenty-three different objective functions for achieving a suitable quasi-optimal solution. These objective functions can be categorized into three different types including: (i) unimodal, (ii) high-dimension multimodal, and (iii) fixed-dimension multimodal functions. Detailed information of these objective functions is given in the Appendix A (Table A1,Table A2 and Table A3).

### 4.1. Experimental Setup

In order to analyze the performance of our proposal, the results obtained by the TSO algorithm are compared, as mentioned, with three classes of existing optimization algorithms, which include (i) GA and PSO, as the most well-studied algorithms (famous methods), (ii) GSA, GWO and TLBO, as algorithms which are cited by many scientists (popular methods), and (iii) MPA, TSA and WOA, as recently developed algorithms (new methods). The experimentation has been done on MATLAB (R2017b version, MathWorks, Natick, MA, USA) using a 64-bit Core i7 processor of 3.20 GHz and 16 GB main memory. For all objective functions, the TSO algorithm and its competing algorithms have been simulated in 20 independent runs, where each run employs 1000 iterations. The optimal solutions of the objective functions are evaluated using the two most important indexes for comparing the performance of algorithms when solving optimization problems, that are: average (AV) and standard deviation (SD) of the best obtained solutions, where, as it is known, such an SD reports the dispersion of these solutions. Indeed, when analyzing the performance of the optimization algorithms with the results presented in Table 1, Table 2 and Table 3, the AV index is important first, but if two algorithms have a similar AV, then the algorithm with less dispersion is superior.

### 4.2. Evaluation for Unimodal Objective Functions

The objective functions *F*_1_ to *F*_7_ are unimodal. The optimization results of the TSO algorithm and other mentioned algorithms for these objective functions are presented in Table 1. For all of these functions, the TSO algorithm performs better than the other eight algorithms. Note that the proposed algorithm provides exactly the global optimal solution for *F*_6_. In addition, for other functions, the TSO algorithm provides a solution very close to the global optimum, especially for *F*_1_ and *F*_2_. These results show that the new proposed algorithm has a good efficiency in achieving a suitable quasi-optimal solution for this type of objective functions.

### 4.3. Evaluation for High-Dimesional Multimodal Objective Functions

Six objective functions *F*_8_ to *F*_13_ are selected from high-dimension multimodal functions. Table 2 reports the results of optimizing these functions using the TSO algorithm and other algorithms. Note that the new algorithm performs better for all *F*_8_ to *F*_13_. Especially for *F*_9_ and *F*_11_, the TSO algorithm has achieved the global-optimal solution. An overview of the results in Table 2 shows that the proposed algorithm is able to solve this type of optimization problems more effectively compared to the other algorithms.

### 4.4. Evaluation for Fixed-Dimesional Multimodal Objective Functions

The functions *F*_14_ to *F*_23_ are used to evaluate the performance of the TSO algorithm and other algorithms for multimodal functions. The results are reported in Table 3. Notice that the new algorithm provides suitable quasi-optimal solutions for this type of functions. Although the MP algorithm also performs well, it is not competitive with the TSO algorithm for *F*_15_, *F*_17_, and *F*_20_. Thus, the new algorithm is more efficient than the other eight algorithms in optimizing this type of objective functions.

**Figure 2:**
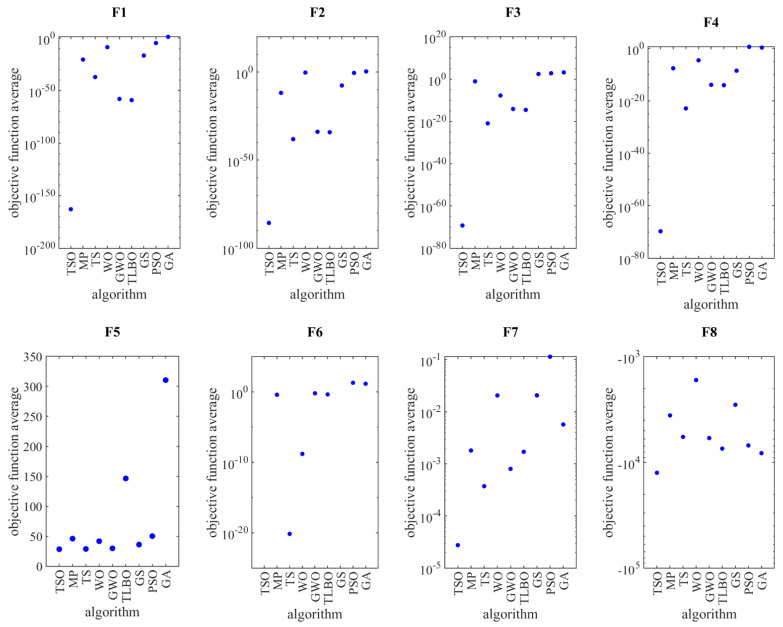

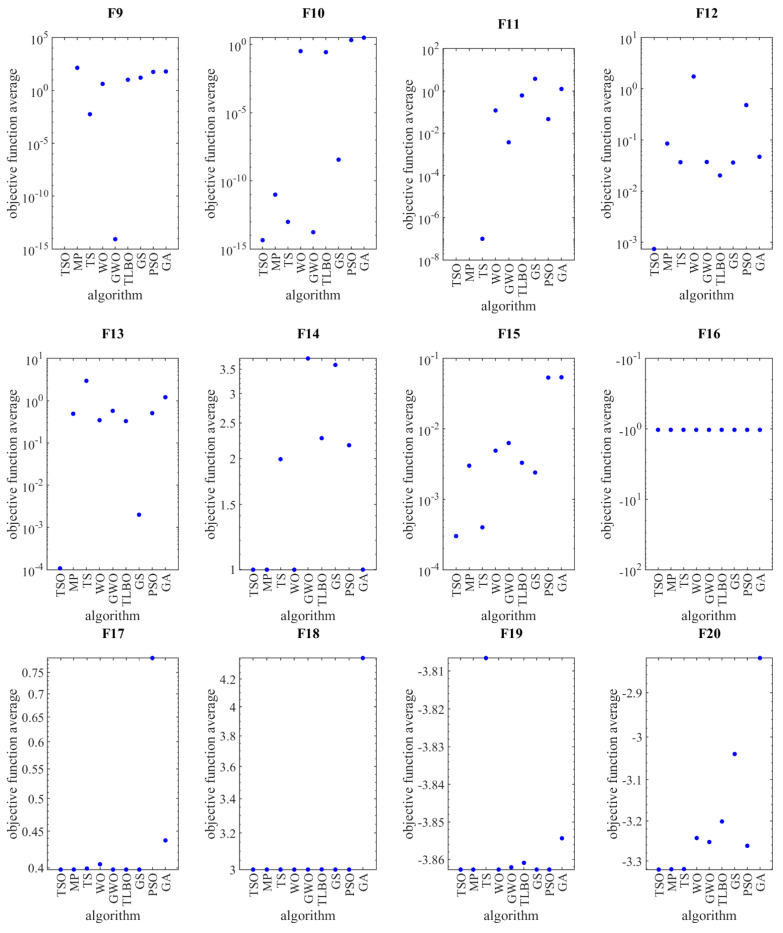

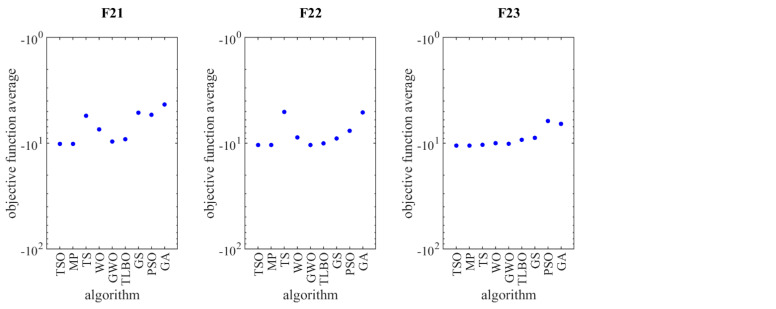
Plots of the objective function average with y-axis in logarithm scale for the indicated algorithm and function.

The AV and SD of the optimal solutions of the objective functions using the proposed TSO algorithm and eight other optimization algorithms are presented in Table 1, Table 2 and Table 3. However, since this class of objective functions are associated with too many local minima, in order to have a better understanding of the results, logarithmic scale plots of the optimal solutions for each algorithm and function are shown in Figure 2.

As mentioned, in order to evaluate the performance of optimization algorithms, objective functions of three different types have been selected. The objective functions *F*_1_ to *F*_7_ of the unimodal type have no local optimum, and the global optimum solution for these functions is zero. Based on the plots of *F*_1_ to *F*_7_, the TSO algorithm provides the best performance among the optimization algorithms. The GA algorithm is the worst optimizer for *F*_1_, *F*_2_, *F*_3_, and *F*_5_. The PSO algorithm is not a good optimizer for *F*_4_, *F*_6_, and *F*_7_. Note that the objective functions *F*_8_ to *F*_13_ are high-dimension multimodal type with local optimal solutions. Considering the plots drawn for these objective functions in Figure 2, it is clear that the TSO algorithm has good performance in solving these types of optimization problems. The distributions of quasi-optimal solutions in the TSO algorithm are very close to each other and therefore have very low SD. The objective functions *F*_14_ to *F*_23_ are fixed-dimension multimodal type with local optimal solutions. The superiority of the TSO algorithm in providing quasi-optimal solutions with low SD is evident in Figure 2 for *F*_14_, *F*_15_, *F*_20_, *F*_21_, *F*_22_, and *F*_23_. As reported in Table 3, the TSO algorithm and other eight algorithms provide similar performance in optimizing the objective functions *F*_16_, *F*_17_, *F*_18_, and *F*_19_. Thus, it is expected that the plots of these functions are similar and practically with no difference to each other.

Based on the analysis of numerical results in Table 1, Table 2 and Table 3 and the plots presented in Figure 2, it is evident that the TSO algorithm is able to provide suitable quasi-optimal solutions with low SD in various problems.

### 4.5. Statistical Testing

Comparison of the performance of the optimization algorithms in providing quasi-optimal solutions based on AV and SD gives us relevant information. However, considering only these results is not enough to guarantee the superiority of an algorithm. This is because, even after twenty independent runs for each algorithm, the superiority of one over the another may occur randomly with very low probability.

Therefore, in order to prove non-randomness superiority of the TSO algorithm, a statistical test on the performance of the algorithms must be considered. In this paper, the Friedman rank test [25], pp. 262–274 is applied for statistical analysis of optimization results and performance of the algorithms. The results of this test for the TSO algorithm and eight other algorithms are reported in Table 4. According to this table, the TSO algorithm ranks first in optimizing unimodal objective functions. After the TSO algorithm, the TSA algorithm ranks second in the optimization of this type of functions. The proposed algorithm also ranks first among eight other algorithms in optimizing high-dimension multimodal objective functions. For this type of functions, after the TSO algorithm, the GWO algorithm ranks second. The proposed algorithm has also achieved the best performance when optimizing fixed-dimension multimodal objective functions. After the TSO algorithm, the MP algorithm is in the second position. In addition, based on general analysis of the results reported in Table 4, for all twenty-three objective functions, the TSO algorithm achieves the best performance among the mentioned optimization algorithms and has the first position. These results confirm the superiority of the TSO algorithm over the other eight algorithms and prove that this superiority is not product of the randomness.

## 5. Discussion

Exploitation and exploration capabilities are two important indicators to evaluate performance of algorithms in providing quasi-optimal solutions [26]. Exploitation power means the ability of an algorithm to achieve a suitable quasi-optimal solution. In fact, at the end of iterations of an algorithm, this must provide the best quasi-optimal solution so far. An algorithm has a higher exploitation power regardless of whether this quasi-optimal solution is closer to the global solution. Exploration power indicates the ability of an optimization algorithm to accurately scan different areas of the search space. Thus, an algorithm that scans the search space more accurately for all iterations can provide a quasi-optimal solution close to the global solution without getting stuck in the local solutions. An important point is to maintain a balance between these two indicators. Then, in the first iterations, the priority is with the exploration index to check the search space well. Therefore, by increasing the number of iterations of the algorithm, the priority is with the exploitation index to achieve the best quasi-optimal solution.

The new TSO algorithm, with suitable number of members, has the potential to accurately scan the search space. Guiding the population members in this space under the influence of several good members causes the population to move to different areas of such a space [27]. This issue increases the ability of the TSO algorithm to accurately scan the search space, which indicates the reasonable exploration power of this algorithm. In addition, as the number of iterations increases, the population members move towards the good members, and as the algorithm reaches the final iterations, population converges and concentrates on near the optimal solution. This issue proves the suitable exploitation power of our TSO algorithm to provide an appropriate quasi-optimal solution.

The analyzed unimodal objective functions have one global optimal solution and no local optimal solutions. Then, these functions are suitable to evaluate the exploitation index. The optimization results of such objective functions presented in Table 1 indicate that the TSO algorithm has an acceptable ability to provide a quasi-solution close to the global solution and has a much higher exploitation power than the other algorithms.

The studied high-dimension and fixed-dimension multimodal functions have several local optimal solutions, in addition to the global optimal solution. Therefore, these types of objective functions are suitable for evaluating the exploration index. Based on the results reported in Table 2 and Table 3, the TSO algorithm, with the desired exploration power, was able to provide appropriate quasi-solutions. This shows that the TSO algorithm has a reasonable ability to accurately scan the search space and therefore has higher exploration power compared to the other eight optimization algorithms.

The statistical results of the Friedman rank test presented in Table 4 confirmed that the superiority of the TSO algorithm over the other eight algorithms analyzed in the exploitation and exploration indexes is not random.

## 6. Conclusions and Future Works

Certain algorithms are able to provide a solution for optimization problems, which is not necessarily the global solution, but could be close to it. In this paper, a two-stage algorithm was introduced to solve optimization problems. The main idea of this algorithm acronymized as TSO is to update the population based on a selected group of its good members. For this purpose, several good members are utilized to lead each population member in all axes of the search space, instead of using only the best member. Therefore, the position of each member in each axis of the search space is updated in two stages and under the influence of two different good members. The main feature of the TSO algorithm is its simplicity of relationships and implementation, as well as the lack of control parameters not needing their tuning.

The stages of the TSO algorithm were described and then mathematically modeled for solving optimization problems. The performance of the proposed algorithm was evaluated on a set of twenty-three objective functions from three different types including unimodal, high-dimension multimodal, and fixed-dimension multimodal functions. The results of this evaluation were compared with the performance of the genetic, gravitational search, grey wolf, marine predators, particle swarm, teaching-learning-based, tunicate swarm, and whale algorithms in optimizing these objective functions [28]. By comparing the simulation results for the unimodal case, which are suitable for evaluating the exploitation index due to having an optimal solution, obvious superiority of the TSO algorithm over the other eight algorithms was demonstrated. Considering the performance of the proposed algorithm and other algorithms on both groups of multimodal objective functions, it was shown that the TSO algorithm has higher exploration power and is superior to other algorithms in optimizing this type of objective functions. Furthermore, the Friedman rank test was applied in order to further analyze the performance of the TSO algorithm and other algorithms. Based on the results of this statistical analysis, it was found that the proposed algorithm ranks first among the studied algorithms and its superiority in optimizing objective functions is not random. Therefore, general analysis of the optimization and statistical results confirmed the superiority of the TSO algorithm doing it more competitive than the other eight analyzed algorithms.

Some ideas and perspectives for future research the arise from the present investigation are the following: (i) the design of the binary version as well as the multi-objective version of the TSO algorithm has an interesting potential; (ii) the implementing of the TSO algorithm on various optimization problems and real-world problems could be explored and achieve some significant contributions [29]; and (iii) it exists a promising area of application in machine, deep and statistical learnings, for instance, in image compression [5]. These and other aspects for further research are being studied by the authors and we hope to publish their finding in future works.

## Figures and Tables

**Table 1:** Results of applying the indicated algorithm on the listed unimodal objective function.

		Genetic	PSO	GS	TLBO	GWO	WO	TS	MP	TSO
*F*_1_	AV	13.2405	1.7740 × 10^−5^	2.0255 × 10^−17^	8.3373 × 10^−60^	1.09 × 10^−58^	2.1741 × 10^−9^	7.71 × 10^−38^	3.2715 × 10^−21^	1.2 × 10^−163^
	SD	4.7664 × 10^−15^	6.4396 × 10^−21^	1.1369 × 10^−32^	4.9436 × 10^−76^	5.1413 × 10^−74^	7.3985 × 10^−25^	7.00 × 10^−21^	4.6153 × 10^−21^	2.65 × 10^−180^
*F*_2_	AV	2.4794	0.3411	2.3702 × 10^−8^	7.1704 × 10^−35^	1.2952 × 10^−34^	0.5462	8.48 × 10^−39^	1.57 × 10^−12^	2.29 × 10^−86^
	SD	2.2342 × 10^−15^	7.4476 × 10^−17^	5.1789 × 10^−24^	6.6936 × 10^−50^	1.9127 × 10^−50^	1.7377 × 10^−16^	5.92 × 10^−41^	1.42 × 10^−12^	1.05 × 10^−99^
*F*_3_	AV	1536.896	589.492	279.3439	2.7531 × 10^−15^	7.4091 × 10^−15^	1.7634 × 10^−8^	1.15 × 10^−21^	0.0864	5.83 × 10^−70^
	SD	6.6095 × 10^−13^	7.1179 × 10^−13^	1.2075 × 10^−13^	2.6459 × 10^−31^	5.6446 × 10^−30^	1.0357 × 10^−23^	6.70 × 10^−21^	0.1444	4.06 × 10^−77^
*F*_4_	AV	2.0942	3.9634	3.2547 × 10^−9^	9.4199 × 10^−15^	1.2599 × 10^−14^	2.9009 × 10^−5^	1.33 × 10^−23^	2.6 × 10^−8^	1.91 × 10^−70^
	SD	2.2342 × 10^−15^	1.9860 × 10^−16^	2.0346 × 10^−24^	2.1167 × 10^−30^	1.0583 × 10^−29^	1.2121 × 10^−20^	1.15 × 10^−22^	9.25 × 10^−9^	4.56 × 10^−83^
*F*_5_	AV	310.4273	50.26245	36.10695	146.4564	36.8607	41.7767	28.8615	46.049	28.4397
	SD	2.0972 × 10^−13^	1.5888 × 10^−14^	3.0982 × 10^−14^	1.9065 × 10^−14^	2.6514 × 10^−14^	2.5421 × 10^−24^	4.76 × 10^−3^	0.4219	1.83 × 10^−15^
*F*_6_	AV	14.55	20.25	0	0.4435	0.6423	1.6085 × 10^−9^	7.10 × 10^−21^	0.398	0
	SD	3.1776 × 10^−15^	1.2564	0	4.2203 × 10^−16^	6.2063 × 10^−17^	4.6240 × 10^−25^	1.12 × 10^−25^	0.1914	0
*F*_7_	AV	5.6799 × 10^−3^	0.1134	0.0206	0.0017	0.0008	0.0205	3.72 × 10^−4^	0.0018	2.75 × 10^−5^
	SD	7.7579 × 10^−19^	4.3444 × 10^−17^	2.7152 × 10^−18^	3.87896 × 10^−19^	7.2730 × 10^−20^	1.5515 × 10^−18^	5.09 × 10^−5^	0.001	8.49 × 10^−20^

Where AV: average and SD: standard deviation.

**Table 2:** Results of applying the indicted algorithm on the listed high-dimension multimodal objective function.

		Genetic	PSO	GS	TLBO	GWO	WO	TS	MP	TSO
*F*_8_	AV	−8184.4142	−6908.6558	−2849.0724	−7408.6107	−5885.1172	−1663.9782	−5740.3388	−3594.16321	−12536.9
	SD	833.2165	625.6248	264.3516	513.5784	467.5138	716.3492	41.5	811.3265	1.30 × 10^−11^
*F*_9_	AV	62.4114	57.0613	16.2675	10.2485	8.5265 × 10^−15^	4.2011	5.70 × 10^−3^	140.1238	0
	SD	2.5421 × 10^−14^	6.3552 × 10^−15^	3.1776 × 10^−15^	5.5608 × 10^−15^	5.6446 × 10^−30^	4.3692 × 10^−15^	1.46 × 10^−3^	26.3124	0
*F*_10_	AV	3.2218	2.1546	3.5673 × 10^−9^	0.2757	1.7053 × 10^−14^	0.3293	9.80 × 10^−14^	9.6987 × 10^−12^	4.44 × 10^−15^
	SD	5.1636 × 10^−15^	7.9441 × 10^−16^	3.6992 × 10^−25^	2.5641 × 10^−15^	2.7517 × 10^−29^	1.9860 × 10^−16^	4.51 × 10^−12^	6.1325 × 10^−12^	7.06 × 10^−31^
*F*_11_	AV	1.2302	0.0462	3.7375	0.6082	0.0037	0.1189	1.00 × 10^−7^	0	0
	SD	8.4406 × 10^−16^	3.1031 × 10^−18^	2.7804 × 10^−15^	1.9860 × 10^−16^	1.2606 × 10^−18^	8.9991 × 10^−17^	7.46 × 10^−7^	0	0
*F*_12_	AV	0.047	0.4806	0.0362	0.0203	0.0372	1.7414	0.0368	0.0851	7.42 × 10^−4^
	SD	4.6547 × 10^−18^	1.8619 × 10^−16^	6.2063 × 10^−18^	7.7579 × 10^−19^	4.3444 × 10^−17^	8.1347 × 10^−12^	1.5461 × 10^−2^	0.0052	1.75 × 10^−18^
*F*_13_	AV	1.2085	0.5084	0.002	0.3293	0.5763	0.3456	2.9575	0.4901	1.08 × 10^−4^
	SD	3.2272 × 10^−16^	4.9650 × 10^−17^	4.2617 × 10^−14^	2.1101 × 10^−16^	2.4825 × 10^−16^	3.25391 × 10^−12^	1.5682 × 10^−12^	0.1932	3.41 × 10^−17^

Where AV: average and SD: standard deviation.

**Table 3:** Results of applying the indicated algorithm on the listed fixed-dimension multimodal objective function.

		Genetic	PSO	GS	TLBO	GWO	WO	TS	MP	TSO
*F*_14_	AV	0.9986	2.1735	3.5913	2.2721	3.7408	0.998	1.9923	0.998	0.998
	SD	1.5640 × 10^−15^	7.9441 × 10^−16^	7.9441 × 10^−16^	1.9860 × 10^−16^	6.4545 × 10^−15^	9.4336 × 10^−16^	2.6548 × 10^−7^	4.2735 × 10^−16^	8.69 × 10^−16^
*F*_15_	AV	5.3952 × 10^−2^	0.0535	0.0024	0.0033	0.0063	0.0049	0.0004	0.003	0.0003
	SD	7.0791 × 10^−18^	3.8789 × 10^−19^	2.9092 × 10^−19^	1.2218 × 10^−17^	1.1636 × 10^−18^	3.4910 × 10^−18^	9.0125 × 10^−4^	4.0951 × 10^−15^	1.82 × 10^−19^
*F*_16_	AV	−1.0316	−1.0316	−1.0316	−1.0316	−1.0316	−1.0316	−1.0316	−1.0316	−1.0316
	SD	7.9441 × 10^−16^	3.4755 × 10^−16^	5.9580 × 10^−16^	1.4398 × 10^−15^	3.9720 × 10^−16^	9.9301 × 10^−16^	2.6514 × 10^−16^	4.4652 × 10^−16^	8.65 × 10^−17^
*F*_17_	AV	0.4369	0.7854	0.3978	0.3978	0.3978	0.4047	0.3991	0.3979	0.3978
	SD	4.9650 × 10^−17^	4.9650 × 10^−17^	9.9301 × 10^−17^	7.4476 × 10^−17^	8.6888 × 10^−17^	2.4825 × 10^−17^	2.1596 × 10^−16^	9.1235 × 10^−15^	9.93 × 10^−17^
*F*_18_	AV	4.3592	3	3	3.0009	3	3	3	3	3
	SD	5.9580 × 10^−16^	3.6741 × 10^−15^	6.9511 × 10^−16^	1.5888 × 10^−15^	2.0853 × 10^−15^	5.6984 × 10^−15^	2.6528 × 10^−15^	1.9584 × 10^−15^	4.97 × 10^−16^
*F*_19_	AV	−3.85434	−3.8627	−3.8627	−3.8609	−3.8621	−3.8627	−3.8066	−3.8627	−3.8627
	SD	9.9301 × 10^−17^	8.9371 × 10^−15^	8.3413 × 10^−15^	7.3483 × 10^−15^	2.4825 × 10^−15^	3.1916 × 10^−15^	2.6357 × 10^−15^	4.2428 × 10^−15^	6.95 × 10^−16^
*F*_20_	AV	−2.8239	−3.2619	−3.0396	−3.2014	−3.2523	−3.2424	−3.3206	−3.3211	−3.3219
	SD	3.97205 × 10^−16^	2.9790 × 10^−16^	2.1846 × 10^−14^	1.7874 × 10^−15^	2.1846 × 10^−15^	7.9441 × 10^−16^	5.6918 × 10^−15^	1.1421 × 10^−11^	1.89 × 10^−15^
*F*_21_	AV	−4.3040	−5.3891	−5.1486	−9.1746	−9.6452	−7.4016	−5.5021	−10.1532	−10.1532
	SD	1.5888 × 10^−15^	1.4895 × 10^−15^	2.9790 × 10^−16^	8.5399 × 10^−15^	6.5538 × 10^−15^	2.3819 × 10^−11^	5.4615 × 10^−13^	2.5361 × 10^−11^	5.96 × 10^−16^
*F*_22_	AV	−5.1174	−7.6323	−9.0239	−10.0389	−10.4025	−8.8165	−5.0625	−10.4029	−10.4029
	SD	1.2909 × 10^−15^	1.5888 × 10^−15^	1.6484 × 10^−12^	1.5292 × 10^−14^	1.9860 × 10^−15^	6.7524 × 10^−15^	8.4637 × 10^−14^	2.8154 × 10^−11^	1.79 × 10^−15^
*F*_23_	AV	−6.5621	−6.1648	−8.9045	−9.2905	−10.1302	−10.0003	−10.3613	−10.5364	−10.5364
	SD	3.8727 × 10^−15^	2.7804 × 10^−15^	7.1497 × 10^−14^	1.1916 × 10^−15^	4.5678 × 10^−15^	9.1357 × 10^−15^	7.6492 × 10^−12^	3.9861 × 10^−11^	9.33 × 10^−16^

**Table 4:** Results of the Friedman rank test for evaluating the indicated algorithm and type of objective function.

Function			TSO	MP	TS	WO	GWO	TLBO	GS	PSO	Genetic
1	Unimodal (*F*_1_–*F*_7_)	Friedman value	7	37	16	42	27	28	37	56	57
		Friedman rank	1	5	2	6	3	4	5	7	8
2	High-dimension multimodal (*F*_8_–*F*_13_)	Friedman value	6	33	27	38	24	25	32	37	40
		Friedman rank	1	6	4	8	2	3	5	7	9
3	Fixed-dimension multimodal (*F*_14_–*F*_23_)	Friedman value	10	15	33	33	31	35	38	45	55
		Friedman rank	1	2	4	4	3	5	6	7	8
4	All 23 functions	Friedman value	23	85	76	113	82	88	107	138	152
		Friedman rank	1	4	2	7	3	5	6	8	9

## Data Availability

The authors declare to honor the Principles of Transparency and Best Practice in Scholarly Publishing about Data.

## Appendix A

Mathematical details of the twenty-three objective functions used for obtaining the results in Table A1, Table A2 and Table A3.

**Table A1:** Unimodal objective functions and their variables’ interval.

Objective Function	Variables’ Interval
$F_{1}\left(x\right)=\overset{m}{\underset{i=1}{\sum }}x_{i}^{2}$	${\left[-100,\;100\right]}^{m}$
$F_{2}\left(x\right)=\overset{m}{\underset{i=1}{\sum }}\left|x_{i}\right|+\overset{m}{\underset{i=1}{\prod }}\left|x_{i}\right|$	${\left[-10,\;10\right]}^{m}$
$F_{3}\left(x\right)=\overset{m}{\underset{i=1}{\sum }}{\left(\overset{i}{\underset{j=1}{\sum }}x_{i}\right)}^{2}$	${\left[-100,\;100\right]}^{m}$
$F_{4}\left(x\right)=\max\left\{\left|x_{i}\right|,\;1\leq i\leq m\right\}$	${\left[-100,\;100\right]}^{m}$
$F_{5}\left(x\right)=\overset{m-1}{\underset{i=1}{\sum }}\left[100{\left(x_{i+1}-x_{i}^{2}\right)}^{2}+{\left(x_{i}-1\right)}^{2})\right]$	${\left[-30,\;30\right]}^{m}$
$F_{6}\left(x\right)=\overset{m}{\underset{i=1}{\sum }}{\left(\left[x_{i}+0.5\right]\right)}^{2}$	${\left[-100,\;100\right]}^{m}$
$F_{7}\left(x\right)=\overset{m}{\underset{i=1}{\sum }}ix_{i}^{4}+\mathrm{rand}\left(0,1\right)$	${\left[-1.28,\;1.28\right]}^{m}$

**Table A2:** High-dimension multimodal objective functions and their variables’ interval.

Objective Function	Variables’ Interval
$F_{8}\left(x\right)=\overset{m}{\underset{i=1}{\sum }}-x_{i}\;\sin\left(\sqrt{\left|x_{i}\right|}\right)$	${\left[-500,\;500\right]}^{m}$
$F_{9}\left(x\right)=\overset{m}{\underset{i=1}{\sum }}\left[\;x_{i}^{2}-10\cos\left(2\pi x_{i}\right)+10\right]$	${\left[-5.12,\;5.12\right]}^{m}$
$F_{10}\left(x\right)=-20\exp\left(-0.2\sqrt{\frac{1}{m}\overset{m}{\underset{i=1}{\sum }}x_{i}^{2}}\right)-\exp\left(\frac{1}{m}\overset{m}{\underset{i=1}{\sum }}\cos\left(2\pi x_{i}\right)\right)+20+e$	${\left[-32,\;32\right]}^{m}$
$F_{11}\left(x\right)=\frac{1}{4000}\overset{m}{\underset{i=1}{\sum }}x_{i}^{2}-\overset{m}{\underset{i=1}{\prod }}\cos\left(\frac{x_{i}}{\sqrt{i}}\right)+1$	${\left[-600,\;600\right]}^{m}$
$F_{12}\left(x\right)=\frac{\pi }{m}\;\left\{10\sin\left(\pi y_{1}\right)+\overset{m}{\underset{i=1}{\sum }}{\left(y_{i}-1\right)}^{2}\left[1+10\sin^{2}\left(\pi y_{i+1}\right)\right]+{\left(y_{n}-1\right)}^{2}\right\}+\overset{m}{\underset{i=1}{\sum }}u\left(x_{i},10,100,4\right),$ where $u\left(x_{i},a,i,n\right)=\{\begin{array}{c} k{\left(x_{i}-a\right)}^{n}\;x_{i}>-a,\\ 0-a<x_{i}<a,\\ k{\left(-x_{i}-a\right)}^{n}\;x_{i}<-a \end{array}$	${\left[-50,\;50\right]}^{m}$
$F_{13}\left(x\right)=0.1\left\{\;\sin^{2}\left(3\pi x_{1}\right)+\overset{m}{\underset{i=1}{\sum }}{\left(x_{i}-1\right)}^{2}\left[1+\sin^{2}\left(3\pi x_{i}+1\right)\right]+{\left(x_{n}-1\right)}^{2}\left[1+\sin^{2}\left(2\pi x_{m}\right)\right]\right\}+\overset{m}{\underset{i=1}{\sum }}u\left(x_{i},5,100,4\right)$	${\left[-50,\;50\right]}^{m}$

**Table A3:** Fixed-dimension multimodal test functions and their variables’ interval.

Objective Function	Variables’ Interval
$F_{14}\left(x\right)={\left(\frac{1}{500}+\overset{25}{\underset{j=1}{\sum }}\frac{1}{j+\sum_{i=1}^{2}{\left(x_{i}-a_{ij}\right)}^{6}}\right)}^{-1}$	${\left[-65.53,\;65.53\right]}^{2}$
$F_{15}\left(x\right)=\overset{11}{\underset{i=1}{\sum }}{\left[a_{i}-\frac{x_{1}\left(b_{i}^{2}+b_{i}x_{2}\right)}{b_{i}^{2}+b_{i}x_{3}+x_{4}}\right]}^{2}$	${\left[-5,\;5\right]}^{4}$
$F_{16}\left(x\right)=4x_{1}^{2}-2.1x_{1}^{4}+\frac{1}{3}x_{1}^{6}+x_{1}x_{2}-4x_{2}^{2}+4x_{2}^{4}$	${\left[-5,\;5\right]}^{2}$
$F_{17}\left(x\right)={\left(x_{2}-\frac{5.1}{4\pi^{2}}x_{1}^{2}+\frac{5}{\pi }x_{1}-6\right)}^{2}+10\left(1-\frac{1}{8\pi }\right)\cos\;x_{1}+10$	[−5, 10]×[0, 15]
$F_{18}\left(x\right)=\left[1+{\left(x_{1}+x_{2}+1\right)}^{2}\left(19-14x_{1}+3x_{1}^{2}-14x_{2}+6x_{1}x_{2}+3x_{2}^{2}\right)\right]\times \left[30+{\left(2x_{1}-3x_{2}\right)}^{2}\times \left(18-32x_{1}+12x_{1}^{2}+48x_{2}-36x_{1}x_{2}+27x_{2}^{2}\right)\right]$	${\left[-5,\;5\right]}^{2}$
$F_{19}\left(x\right)=-\overset{4}{\underset{i=1}{\sum }}c_{i}\exp\left(-\overset{3}{\underset{j=1}{\sum }}a_{ij}{\left(x_{j}-P_{ij}\right)}^{2}\right)$	${\left[0,\;1\right]}^{3}$
$F_{20}\left(x\right)=-\overset{4}{\underset{i=1}{\sum }}c_{i}\exp\left(-\overset{6}{\underset{j=1}{\sum }}a_{ij}{\left(x_{j}-P_{ij}\right)}^{2}\right)$	${\left[0,\;1\right]}^{6}$
$F_{21}\left(x\right)=-\overset{5}{\underset{i=1}{\sum }}{\left[\left(X-a_{i}\right){\left(X-a_{i}\right)}^{T}+6c_{i}\right]}^{-1}$	${\left[0,\;10\right]}^{4}$
$F_{22}\left(x\right)=-\overset{7}{\underset{i=1}{\sum }}{\left[\left(X-a_{i}\right){\left(X-a_{i}\right)}^{T}+6c_{i}\right]}^{-1}$	${\left[0,\;10\right]}^{4}$
$F_{23}\left(x\right)=-\overset{10}{\underset{i=1}{\sum }}{\left[\left(X-a_{i}\right){\left(X-a_{i}\right)}^{T}+6c_{i}\right]}^{-1}$	${\left[0,\;10\right]}^{4}$
